# Annotations

**Published:** 1894-06-23

**Authors:** 


### ANNOTATIONS.

### Can Doctors Prevent Insanity?

In a very able article on the question of the increase of insanity in Ireland, the *Times* tells us that "The Ancients represented insanity as a power sent by the gods to vex and perturb mortals who had offended them. . . We do not seem to have got any clearer idea of the causes of insanity." If the *Times* be right, then the question at the head of this Annotation is a very foolish one. But the *Times* is not right. We make bold to say that the prevention of insanity is well within the power of medical science, speaking in the large sense. What is not within the power of medical science is the opportunity of applying itself to this great work. Here is the argument in a sentence: Prevent the marriage of those who have congenitally insane tendencies; make the conditions of life reasonably happy for the masses of the people; diet the people according to physiological reason, and the thing is done. No doubt the flippant person will say, "thank you for nothing." But we submit that the question, What medical science can do when it has an entirely free hand is one thing; the question what it can do when embarrassed by all sorts of politico-economical impedimenta is quite another. We do not ask medical science to cure a broken leg whilst the patient is attempting to walk six miles a day. We should be fools if we did. Why, then, should we ask medical science to prevent insanity, when we deny it the power of putting a check upon the causes of insanity? We maintain that medical science has done its part, and a very noble part, when it has put its finger upon the causes of insanity with absolute and demonstrated certainty. When the public is wiser, if it is destined ever to be any wiser, it will acknowledge the great merit of this part of medical science; and, what will be far better, it will, as much as possible, help to clear the ground for the universal application of that science in daily life.

### Unconscious Self-Poisoning.

Dr. William Carter, of Liverpool, anticipates what we feel sure will constitute an important part of the medicine of the future, in a small pamphlet, which he has done well to publish, and which records a series of most interesting cases of auto-infection of a dangerous and deadly character. One case is typical of the series. It may be summarised in a very few sentences. A Liverpool clergyman found his health gradually failing without manifest cause. He lost strength, and his memory began to be the subject of brief lapses in the pulpit. He was advised to travel. On his travels he became feverish; vomited occasionally, with streaks of blood in the vomit; grew exceedingly weak; showed marked symptoms of jaundice; and was considered by one foreign medical man to be suffering from cancer of the liver. At a later stage intermittent fever of a hectic character showed itself; the strength was reduced to a minimum; emaciation was extreme; the jaundice continued; the appetite failed; and patient, doctors, and friends all considered that death was imminent. What was the cause of all these apparently inexplicable symptoms? For many weeks no cause was discovered. At last attention was called to the nose. There had been noticed occasional bleedings, with an offensive discharge; ulceration was suspected; antiseptic flushing was commenced and carried out; and one by one all the symptoms disappeared, and the patient regained perfect health. If this case had stood alone, "coincidence" might have been objected by the sceptical. But Dr. Carter records no fewer than five such cases. There will be no hesitation on the part of those medical men who are also physiologists, in accepting Dr. Carter's conclusions, even though the data are somewhat limited. What we desire to point out is the new kind of vista which is opened out to the mind of the inquiring practitioner whose physiology is no longer actively fresh in his mind. The one conclusion to be drawn is that the possibilities of auto-infection are innumerable. Not merely the nose, but the throat, the rectum, and other explorable parts may be the seats of lesions which produce poisons capable of absorption; and not only those parts, but every structure of the body, hidden as well as patent, may be the seat of structural disintegrations, and the source of auto-infections which give rise to symptoms explicable on no other hypothesis. Want of space does not permit us to enlarge; but the field of exploration is comparatively new, at any rate in common practice. Carefully conducted investigation, with the intelligent use of local and internal antiseptics, on the lines of Dr. Carter's pamphlet, may result in the definite diagnosis of many mysterious complaints, and the happy restoration of the patients to health. Every practitioner should make a point of reading Dr. Carter's most practical and interesting monograph.

### Medical Protection.

Under the presidency of Mr. Jonathan Hutchinson, F.R.S., the London and Counties Medical Protection Society held its annual meeting and dinner on Tuesday at the Restaurant Frascati, in New Oxford Street. The society announces a steady increase in the number of its members and a solvent exchequer. It was stated by the officers, and emphasised by Mr. Hutchinson, that the prosecution of the so-called "Indian oculists" during the financial year had been generally approved by the press and the public, and had been of substantial utility both to the general community and the medical profession. Nevertheless, it had proved a costly experiment, and had used up resources which were needed for the more immediately necessary objects of medical defence. The case of Dr. Maxwell, which had been referred by the Council to the annual meeting, was considered at length. Dr. Maxwell, it will be remembered, had been prosecuted by Dr. Logie for alleged libel, and a verdict, with damages amounting to one farthing, had been obtained against him. In considering the case at the time of its occurrence, the Council of the Society came to the conclusion that Dr. Maxwell, though morally in the right, had by his words and actions put himself technically in the wrong, and that, therefore, the rules of the Society did not permit of his defence by the Council. Under these unusual circumstances the Council asked the annual meeting to make a grant towards Dr. Maxwell's costs, and the sum of £86 was voted. A very pleasant dinner brought a very successful meeting to a close.

